# Artificial intelligence, diagnostic imaging and neglected tropical diseases: ethical implications

**DOI:** 10.2471/BLT.19.237560

**Published:** 2020-03-03

**Authors:** Alon Vaisman, Nina Linder, Johan Lundin, Ani Orchanian-Cheff, Jean T Coulibaly, Richard KD Ephraim, Isaac I Bogoch

**Affiliations:** a Division Infectious Diseases, Toronto General Hospital, University of Toronto, 14EN 209, 200 Elizabeth Street, Toronto, Ontario M5G 2C4, Canada.; b Department of Women's and Children's Health, International Maternal and Child health, Uppsala University, Sweden; c Department of Global Public Health, Karolinska Institutet, Stockholm, Sweden; d Health Sciences Library, University Health Network, Toronto, Canada.; e Unité de Formation et de Recherche Biosciences, Université Félix Houphouët-Boigny, Abidjan, Côte d'Ivoire.; f Department of Medical Laboratory Sciences, University of Cape Coast, Cape Coast, Ghana.

Artificial intelligence, defined as a system capable of interpreting and learning from data to produce a specific goal,^1^ has made significant advances in the field of neglected tropical diseases. Specifically, artificial intelligence is increasingly applied to the task of interpreting images of such diseases and generating accurate and reliable diagnoses that may ultimately inform management of these conditions. Neglected tropical diseases affect over a billion people globally and are a significant source of morbidity and mortality in low- and middle-income countries.^2^ Artificial intelligence has the potential to transform how such diseases are diagnosed and may contribute to enabling clinical and public health delivery in low- and middle-income countries. For example, artificial intelligence applied to neglected tropical disease diagnosis may help drive point-of-care clinical decision-making, identify outbreaks before they spread and help map these diseases to guide public health surveillance and control efforts. The latest research in this field demonstrates that novel diagnostic tools, such as mobile phone microscopes have rapidly improved diagnostic characteristics and broadened the scope of pathogens tested, and have excellent functionality in neglected tropical disease-endemic settings.^3^^,^^4^ Such devices are already being field tested and implemented on a limited scale, for example in Côte d’Ivoire.^5^

However, careful consideration to several ethical concerns arising from artificial intelligence-driven diagnoses of neglected tropical diseases in low-resource settings is critical for maximizing the benefit of this technology.^6^ Artificial intelligence applications focused on image-based diagnoses is still in its infancy and therefore, now is an opportune time to ensure that these applications develop within an ethical framework. Here, we outline important ethical challenges faced by low- and middle-income countries that may benefit from the implementation of these technologies. Key issues discussed include the interrelationships between stakeholder engagement, consent, data security, accessibility of technology, adhering to current and evolving care standards and deciding how to effectively use resources. Addressing these issues during the design phase of artificial intelligence technology will facilitate its timely implementation and maximize public health benefit.

Most published studies focusing on the development of artificial intelligence tools for image-based diagnoses are conducted in laboratories based in high-income countries. Consequently, the limited engagement of scientists and clinicians from endemic regions may restrict the utility, and eventually scale, of these technologies in precisely the countries that would benefit the most. Therefore, several stakeholders should be involved from the earliest phases in the development of artificial intelligence tools.^7^ These stakeholders include data scientists and engineers from both low- and middle-income countries affected by neglected tropical diseases and those from high-income countries currently working in artificial intelligence diagnostics. Pairing teams of data scientists and engineers would enable capacity building in low- and middle-income countries where there is currently limited infrastructure to develop such diagnostic tools. Other important stakeholders include clinical, public health, governmental and citizen representation from low- and middle-income countries affected by these diseases. Such groups are critical in the identification of priority areas, shaping research questions and implementing the technology into routine health-care use. Private industry and governmental bodies should also be instrumental in the scale-up, licensing and regulation of new diagnostic tools, and their involvement and support during concept development may help streamline product development.^8^

Addressing ethical issues surrounding informed consent, an issue closely intertwined with data security, is vital in the development of artificial intelligence image-based diagnostic tools for neglected tropical diseases. Diagnostic tests inherently involve some form of biologic sample collection from a patient and this procedure is frequently connected to patient-identifying information. Although these diagnoses may be performed at the point of care,^9^ the collected specimens and images may be subsequently used to train and improve machine-learning algorithms. Individuals providing samples must consent to their biologic sample, and perhaps other personal data. Similarly, individuals must be notified of which personal information is being used and stored, where it is being stored, who has access to this data, how it is being accessed and how this personal information is being used or may be used in the future.^1^^,^^6^ Given that much of this diagnosis technology has been developed in high-income countries for use in low- and middle-income countries, special attention is required. Therefore, the informed consent process in low- and middle-income countries must adhere to the highest standards that are respectful and inclusive of culture, language, religion, gender, age and socioeconomic status.^10^

Ethical concerns may also arise due to the accessibility of artificial intelligence technology for neglected tropical disease diagnoses. Since this technology can be transformative to communities burdened by such diseases, its accessibility to all affected populations, including to those in underserviced and remote communities, must be insured. Early and broad stakeholder engagement in project development can ensure that artificial intelligence diagnostics tools tailored for low- and middle-income countries will be accessible and barriers, such as affordability and scale will be considered in countries burdened by these diseases. Examples of mitigating access issues include facilitating the development and use of open-source software in the early stages of product development to help lower implementation costs and share information.^11^

We must also examine how these technologies affect the standards of care as both the technology developed and local care standards will continue to evolve. While artificial intelligence-based diagnoses of neglected tropical diseases may evolve into preferred methods for diagnosis, further advances in this field and others will inevitably take place, and appropriate regulation and oversight will be essential to ensure that diagnostic tools continue to adhere to local standards of care.^12^ Hence, early and continuous involvement of government, industry and health-care teams is essential for the continuous maintenance of diagnostic quality. Furthermore, as these technologies continue to evolve, low- and middle-income countries must not be left behind as image-based artificial intelligence diagnostic tools improve and expand in the health-care sector.

Lastly, there may be unintended consequences for implementing these diagnostic tools in low- and middle-income countries. While most intentions seem to be laudable, such as facilitating care in low-resource settings, many of these countries have significant limitations in health-care infrastructure and challenges in the provision of health care to large segments of their population. The implementation of artificial intelligence diagnostics could unintentionally draw vital resources from other programmes. Ensuring broad engagement from the outset may help mitigate these issues by identifying priority areas specific to particular countries. Stakeholders can conduct current-state assessments for future innovations to determine the impact, both positive and negative, of artificial intelligence diagnostic implementation, and decide how and if such innovations can be used locally and the appropriate timing of implementation.

Image-based artificial intelligence for the diagnoses of neglected tropical diseases has the potential to transform health care in low- and middle-income countries affected by these diseases. While this field is still in its early stages, there is potential to bring quality diagnostic tools to clinical and public health settings in the most underserved regions. As this field evolves, integrating an ethical framework for the development of these tools will enable their sustainability and utility. Broad stakeholder engagement, a focus on consent and data security, and balancing the use of limited public health resources are important principles that can be introduced early in the development of this technology. Doing so will ensure the most impactful use of artificial intelligence diagnoses for neglected tropical diseases and its ultimate long-term success and sustainability.
